# Paternally Transmitted Mitochondria Express a New Gene of Potential Viral Origin

**DOI:** 10.1093/gbe/evu021

**Published:** 2014-02-05

**Authors:** Liliana Milani, Fabrizio Ghiselli, Maria Gabriella Maurizii, Sergey V. Nuzhdin, Marco Passamonti

**Affiliations:** ^1^Dipartimento di Scienze Biologiche, Geologiche ed Ambientali, University of Bologna, Italy; ^2^Program in Molecular and Computational Biology, Department of Biological Sciences, University of Southern California

**Keywords:** mitochondrial ORFan, viral endogenization, novel mitochondrial protein, testis expression, doubly uniparental inheritance of mitochondria, embryo development

## Abstract

Mitochondrial ORFans (open reading frames having no detectable homology and with unknown function) were discovered in bivalve molluscs with doubly uniparental inheritance (DUI) of mitochondria. In these animals, two mitochondrial lineages are present, one transmitted through eggs (F-type), the other through sperm (M-type), each showing a specific ORFan. In this study, we used in situ hybridization and immunocytochemistry to provide evidence for the expression of *Ruditapes philippinarum* male-specific ORFan (*orf21*): both the transcript and the protein (RPHM21) were localized in spermatogenic cells and mature spermatozoa; the protein was localized in sperm mitochondria and nuclei, and in early embryos. Also, in silico analyses of *orf21* flanking region and RPHM21 structure supported its derivation from viral sequence endogenization. We propose that RPHM21 prevents the recognition of M-type mitochondria by the degradation machinery, allowing their survival in the zygote. The process might involve a mechanism similar to that of Modulators of Immune Recognition, viral proteins involved in the immune recognition pathway, to which RPHM21 showed structural similarities. A viral origin of RPHM21 may also support a developmental role, because some integrated viral elements are involved in development and sperm differentiation of their host. Mitochondrial ORFans could be responsible for or participate in the DUI mechanism and their viral origin could explain the acquired capability of M-type mitochondria to avoid degradation and invade the germ line, that is what viruses do best: to elude host immune system and proliferate.

## Introduction

One intriguing observation that emerged from sequencing of whole mtDNAs is the occurrence of numerous ORFans (open reading frames having no detectable homology and with unknown function) showing homologies in closely related species only. Interestingly, some of these genes were shown to be involved in key biological functions (Monchois et al. 2001; Khalturin et al. 2008, 2009). For example, novel mtDNA-encoded proteins are known to act on gamete formation in angiosperm plants exhibiting cytoplasmic male sterility (CMS) (Chase 2007). Novel lineage-specific mitochondrial ORFs (Breton et al. 2009, 2011a, 2011b; Ghiselli et al. 2013) were also discovered in animals with doubly uniparental inheritance (DUI) of mitochondria (Skibinski et al. 1994a, 1994b; Zouros et al. 1994a, 1994b). In metazoans, mitochondria are commonly inherited by strictly maternal inheritance (SMI) (Birky 2001), but, in some bivalve molluscs, two mitochondrial lineages are present, one transmitted through eggs (F-type) and the other through sperm (M-type).

All the analyzed DUI species show the following two interesting features: 1) The existence of sex-biased progeny was observed in species of the *Mytilus edulis* complex (Saavedra et al. 1997; Kenchington et al. 2002; Cogswell et al. 2006), and recently also in *Ruditapes philippinarum* (Ghiselli et al. 2012). The sex bias appears to be controlled by the nuclear genome of the females which can be accordingly subdivided in three classes: those producing predominantly males, those producing predominantly females, and those producing females and males at intermediate frequencies. 2) In embryos of DUI species the distribution of sperm mitochondria follows two different patterns: aggregated or dispersed (Cao et al. 2004; Obata and Komaru 2005; Cogswell et al. 2006; Milani et al. 2011, 2012). In *Mytilus,* it was possible to correlate the pattern type to the sex of the embryo: male-biased offsprings showed a prevalence of aggregated patterns, while female-biased offsprings a prevalence of dispersed patterns. It was then proposed that in developing males the aggregate of sperm mitochondria enters the primordial germ cells (PGCs), providing an early mechanism through which sperm mitochondria are segregated into the germ line of male embryos; after that, the M mitochondrial genome becomes dominant in male germ line, as indicated by the exclusive presence of M-type mtDNA in spermatozoa (Venetis et al. 2006; Ghiselli et al. 2011). In DUI female embryos, sperm mitochondria are dispersed, diluted or possibly degraded, and the mitochondrial inheritance would be as in SMI species.

The peculiar segregation pattern of spermatozoon mitochondria, correlated with the existence of sex-biased lineages, led to the hypothesis that M-type mitochondria may have an active role in gonad masculinization, achieved through a series of specific signals between nucleus and mitochondria (Kenchington et al. 2002). This is not unthinkable, because the involvement of mitochondria in germ line development is well established. For example, in *Drosophila*, mitochondrial ribosomes are found extramitochondrially and are required to produce proteins that are necessary for germ cell formation (Amikura et al. 2001 and references therein). Actually, in early embryos, a mitochondrial-type translation is required for germ cell formation, which is disrupted by the injection of prokaryotic translation inhibitors (Amikura et al. 2005). Moreover, translation of nuclear-encoded proteins by mitochondrial-type ribosomes was detected in mammalian sperm (Gur and Breitbart 2006).

The molecular mechanisms underlying DUI are still unknown; however, many structural and functional features of M and F mtDNAs were proposed as candidates for a role in mitochondrial inheritance and germ line establishment/differentiation (Ghiselli et al. 2013; Milani, Ghiselli, Guerra, et al. 2013; Zouros 2013). Among these candidates, novel lineage-specific ORFs found in DUI species belonging to the families Unionidae (Breton et al. 2009, 2011a), Mytilidae (Breton et al. 2011b), and Veneridae (Ghiselli et al. 2013) were proposed. So far, the existence of the translation product was verified in the unionid *Venustaconcha ellipsiformis* only (Breton et al. 2009, 2011a). Milani, Ghiselli, Guerra, et al. (2013) used multiple in silico approaches to perform a comparative analysis of DUI mitochondrial ORFans and proposed their origin through viral endogenization.

In the DUI species *R. philippinarum*, the novel lineage-specific ORFs are localized in different position in the two mitochondrial genomes. The F-specific ORF is located in the largest unassigned region of the F-type mtDNA and is not present in the M-mtDNA, while the M-specific ORF is located in the Unassigned Region 21 (UR21) of the M-type mtDNA, and is not present in the F-mtDNA. Transcriptomic data (Ghiselli et al. 2013) showed that, while the F-specific ORF is poorly transcribed compared with the mitochondrially encoded electron transport chain (ETC) genes, the M-specific ORF is transcribed at the same level of ETC genes. Moreover, while the F-specific ORF shows a few nonsynonymous substitutions (Ghiselli et al. 2013), the amino acid sequence of M-specific ORF is 100% conserved in all analyzed males. These findings suggest M-specific ORF functionality. This study is focused on the M-specific ORF, which will be here referred to as *orf21*. We performed an in-depth analysis on *orf21* transcript and on its product, RPHM21 (from *R. philippinarum* Male mitochondrial protein 21): we localized *orf21* transcripts by in situ hybridization (ISH), and synthesized a specific antibody to verify the existence, by Western blotting and confocal microscopy, of RPHM21 in adults and early embryos. Moreover, we performed additional analyses of *orf21* flanking region and RPHM21 structure to further support its possible viral derivation and to find similarity with known proteins. We discussed the putative function of RPHM21, its role in DUI and embryonic development, its origin and the possibility that such novel gene was co-opted acquiring important functions in *R. philippinarum* development.

## Materials and Methods

### *orf21* Transcript Localization with ISH

ISH analysis was performed on *R. philippinarum* gonads and surrounding tissues of five specimens, three males and two females. Overall, we processed and checked 60 sections. An antisense probe against the strictly conserved novel mitochondrial ORF in the Male Unassigned Region 21 (MUR21) of M-type mtDNA was produced. The probe, containing the entire *orf21* sequence of 519 bp, was generated from the M-type mitochondrial whole genome sequence present in GenBank (AB065374). The primers used were *orf21*-forward 3′-GGTAGCACAAGGTTTCCAGAGTTTATGTGT-5′, and *orf21*-reverse 3′-ACTTGTAAACCAGGGGTAAGAGGTCACA-5′. The T7 RNA polymerase-binding sequence was added 5′ to the antisense DNA sequence. RNA extraction was performed by TRIzol RNA Isolation Reagents (Life Technologies). The target sequence was amplified by PCR from cDNAs obtained by SuperScript III First-Strand Synthesis System for RT-PCR (Invitrogen – Life Technologies). A Digoxigenin (DIG)-labeled riboprobe was obtained using the Roche in vitro transcription labeling protocol (Roche DIG RNA labeling kit). ISH was performed on 10–20 µm cryosectioned sections, following the method in (Milani, Ghiselli, Nuzhdin, et al. 2013). Samples were analyzed with a Nikon Eclipse 80i microscope and images were captured using NIS-Elements D3.2 software.

### Spawning Induction and Fertilization

*Ruditapes philippinarum* clams used for mating were collected during the reproductive season (July/August 2012). The spawning was induced with 30-min cycles of cold (22 °C) and warm (29 °C) artificial sea water (i.e., reverse osmosis water added with RedSea Coral Pro aquariology sea salt). Two matings were performed: two females were fertilized with sperm from the same male. After fertilization, embryo development was visually checked with an optical microscope. Embryo samples were collected at 40–45 min, 60–65 min, and 2 h, timing that corresponds to 2, 4–8, and 16–32 blastomere stages (see Milani et al. 2012).

### SDS-PAGE and Western Blotting

Pieces of male and female gonads were frozen in liquid nitrogen, stored at −80 °C, and then processed by SDS-PAGE (sodium dodecyl sulphate–polyacrylamide gel electrophoresis) and Western blotting. Samples were homogenized in RIPA buffer (Tris–HCl 50 mM, pH 7.4, added with 150 mM NaCl, 1% NP-40, 0.25% Na-deoxycholate, 1 mM EDTA) in the presence of protease inhibitor cocktail tablets (Complete Mini, Roche) and 1 mM PMSF, utilizing an Ultra Turrax T25 Janke & Kunkel IKA-labortechnik. Next, samples were centrifuged 10 min at 4 °C at 10,000 rpm. The supernatant was treated with 0.25–0.6 U/µl of DNase I (from bovine pancreas; Sigma-Aldrich) and 0.004–0.009 U/µl of RNase A (from bovine pancreas; Sigma-Aldrich) in a 50 mM MgCl_2_ solution, followed by 30 min incubation on ice, to remove nucleic acids that prevented a correct running during electrophoresis.

Gonadic extracts of *R. philippinarum* (60 µg) were separated in 12% SDS polyacrylamide gels according to (Laemmli 1970). For immunoblotting, proteins were transferred to Hybond-ECL membrane (Amersham International, Buckinghamshire, UK). Nonspecific protein-binding sites were blocked with 5% dried skimmed milk (Bio-Rad Laboratories, Hercules, CA, USA), 3% bovine serum albumin (BSA), and 0.1% Tween-20 (Sigma) in TBS 1 h 30 min at RT, and subsequently washed with 0.1% Tween TBS. To recognize RPHM21 protein, we utilized a specific antiserum produced in rabbit (anti-RPHM21; Davids Biotechnologie), diluted 1:80,000 with 0.1% Tween TBS, overnight at 4 °C. After rinsing, we incubated with goat anti-rabbit secondary antibody conjugated with horseradish peroxidase (Santa Cruz Biotechnology Inc., Santa Cruz, CA, USA) at the dilution of 1:5,000 for 1 h 20 min at RT. The washed membranes were detected with ECL Western Blotting Detection Reagents (Roche) and exposed to Hyperfilm ECL (GE Healthcare). 

RPHM21 expected weight was calculated from its amino acid sequence (http://www.bioinformatics.org/sms/prot_mw.html, last accessed February 10, 2014).

### *orf21* Protein (RPHM21) Localization: Tissue Processing and Immunocytochemical Analysis

#### Embryos

Embryos were fixed in a solution containing 3.7% paraformaldehyde and 0.1% glutaraldehyde, in K-PIPES buffer (80 mM K Pipes; 1 mM MgCl_2_; 5 mM EGTA; 0.2% Triton X-100) (pH 6.8) for 30 min at 37 °C. After several washes in Tris-buffered saline solution (TBS: 155 mM NaCl; 10 mM Tris–HCl) (pH 7.4), embryos were put in 100% methanol and stored at 4 °C. After rehydration with TBS (pH 7.4), fixed embryos were put on slides coated with APES (Sigma), treated with 50 mM sodium borohydride in TBS for 60 min at RT and rinsed in TBS with several changes. Next, embryos were treated with 0.01% Pronase E (Merck) in phosphate-buffered saline solution (PBS) (128 mM NaCl; 2 mM KCl; 8 mM Na_2_HPO_4_·2H_2_O; 2 mM KH_2_PO_4_) (pH 7.2), for 10–12 min at RT. Permeabilization was performed adding TBS-Triton 1% to all the samples and leaving over night at 4 °C.

#### Gonads

Pieces of gonads were fixed in 3.7% paraformaldehyde and 0.1% glutaraldehyde solution in K-PIPES buffer (pH 7) for 3 h and embedded in 7% agar. Sections of about 100-µm thickness were made using a Lancer Vibratome Series 1000 and post-fixed with increasing concentrations of methanol. Sections were rehydrated and then treated 1 h and 15 min with sodium borohydride 70 mM in TBS (pH 7.4) at RT. After rinsing 1 h and 15 min in TBS-Triton 0.1%, sections were treated with 0.01% Pronase E, as above, but for 18 min at RT. After, samples were permeabilized adding TBS-Triton 1% and left over night at 4 °C.

Nonspecific protein-binding sites in both gonads and embryos were blocked with a buffer containing 10% normal goat serum (NGS) and 1% BSA in TBS 0.1% Triton (pH 7.4). Sections were incubated with anti-RPHM21 diluted 1: 8,000 with TBS containing 0.1% Triton and 3% BSA (pH 7.3) for 48 h at 4 °C. After washing (several changes in about 8 h), sections were incubated with secondary antibody, a goat anti-rabbit polyclonal antibody, conjugated with *N*,*N*′-(dipropyl)-tetramethylindocarbocyanine (Cy3) (Zymed, Molecular Probes), diluted 1:250 with TBS containing 0.1% Triton, 10% NGS, and 1% BSA (pH 7.4) for 30 h at 4 °C.

Nuclei were stained with 0.5 or 1 μM TO-PRO3 (Molecular Probes), for embryos and gonadic sections, respectively, in PBS (pH 7.2) at RT for about 2–3 min. Samples were mounted in 2.5% 1,4-diazabicyclo[2.2.2]octane (DABCO; Sigma), 50 mM Tris (pH 8) and 90% glycerol, and stored at 4 °C. 

Controls were performed using samples in which the first or the second antibody was omitted, or samples treated only with normal serum. Moreover, in additional control samples, the synthetic peptides used in antibody production were added to the primary antibody solution at a ten-fold concentration, finally accessing the decreasing staining intensity and confirming the reaction specificity (the abcam protocol was utilized as method to block with immunizing peptide). Imaging was recorded by a confocal laser scanning microscope (Leica confocal SP2 microscope), using Leica software.

### Structure and Function Analyses

The *orf21* translation (putative protein RPHM21; from the M-type complete mitochondrial genome; GenBank: AB065374) was analyzed with several programs. Given the ORFan status of RPHM21, we needed to find signatures of short sequences and remote homology, so we used tools that were specifically developed for these tasks. ProTeus (http://www.proteus.cs.huji.ac.il/, last accessed February 10, 2014; Bahir and Linial 2005) detects previously known and potentially overlooked signatures in protein termini. It focuses on signatures that are often undetected by most search methods due to an inadequate statistical significance score that fails to recognize very short signatures (Bahir and Linial 2005). HMMER (http://hmmer.janelia.org/search/phmmer, last accessed February 10, 2014; Finn et al. 2011) searches sequence databases for protein homologs using profile hidden Markov models. Compared with other search tools based on the classic scoring methodology, it is more accurate and more able to detect remote homologs (Finn et al. 2011). FUGUE (http://www-cryst.bioc.cam.ac.uk/fugue/, last accessed February 10, 2014; Shi et al. 2001) uses sequence-structure comparison to recognize distant homologs.

To predict protein secondary structure, we used Quick2D (http://toolkit.tuebingen.mpg.de/quick2_d, last accessed February 10, 2014): it finds secondary structure features like alpha helices, extended beta sheets, coiled coils, transmembrane helices (TMHs), and disorder regions. The advantage of this tool is that it combines the results of up to 11 different structure prediction algorithms (see website for details). Quick2D was used to predict protein secondary structure of both RPHM21 and MK3, an E3 ubiquitin ligase (also named MIR1 of [Murid herpesvirus 4], NCBI Reference Sequence: NP_044852.1) to which many function similarities were found with RPHM21.

Since structural predictions revealed the putative presence of transmembrane domains in both RPHM21 and MK3, we used TM-Coffee (http://tcoffee.crg.cat/apps/tcoffee/do:tmcoffee, last accessed February 10, 2014; Chang et al. 2012) to produce their alignment. To obtain a model for protein transmembrane topology, we used PredictProtein (http://www.predictprotein.org/, last accessed February 10, 2014; Rost et al. 2004), which utilizes 30 different methods (see Rost et al. 2004 for details) of structural prediction. To infer the subcellular localization, we also used LocTree2 (https://www.rostlab.org/services/loctree2/, last accessed February 10, 2014; Goldberg et al. 2012) that is particularly efficient in de novo prediction. In general, given the absence of RPHM21 detectable homologs in the databases, we decided to use tools able to detect remote homology and to predict protein features de novo or by structural similarity. Moreover, we favored multialgorithm approaches (and combinations of them), because we feel that getting converging results from several different methods makes the derived assumptions stronger, strengthening the results.

TOPO2 was used to obtain a protein structure representation (http://www.sacs.ucsf.edu/TOPO2/, last accessed February 10, 2014). An analysis of the whole MUR21 nucleotide sequence was performed with BlastN. All databases were accessed by August 2013.

## Results

### Localization of *orf21* Transcripts and RPHM21 Protein

**Fig. 1.—:**
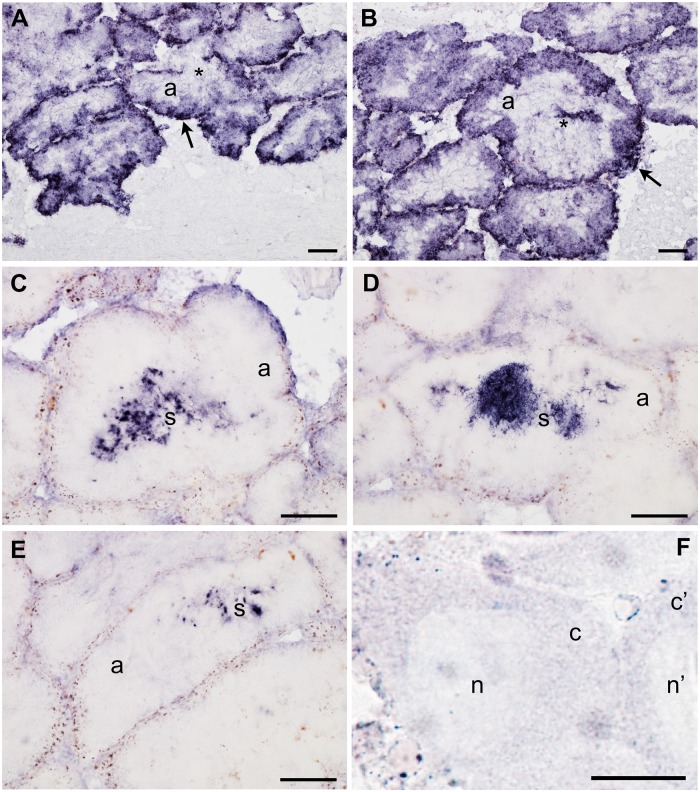
*orf21* transcript localization with ISH in male and female gonadic tissue of *Ruditapes philippinarum*. (*A–E*) male gonadic tissue staining (a = acinus = gonadic unit): (*A*) in the immature acinus, the probe labeled the acinus periphery, indicating a positive reaction with spermatogenic cells, while the acinus center (asterisk), where a lumen will form after sperm maturation, is not stained; (*B*) mature spermatozoa begin to be stored in the acinus center during the lumen formation (asterisk). *orf21* transcript appears to be more present (higher staining) along the acinus wall (arrow in *A* and *B*); (*C–E*) mature acini with the lumen full of stained mature spermatozoa (s) (well evident in *D*). (*A–E* scale bars = 100 µm); (*F*) no staining is present in *R. philippinarum* eggs (n = egg nucleus; c = egg cytoplasm) (F scale bar = 25 µm). Positive signal with antisense probe hybridization stained dark blue.

ISH with *orf21* antisense probe in *R. philippinarum* male gonad showed a specific transcription in both the acinus (gonadic unit containing germinal cells) and its lumen (the cavity of the acinus that is full of gametes at maturity), indicating a positive reaction with both spermatogenic cells (fig. 1*A* and *B*) and mature spermatozoa that are free in the lumen (fig. 1*C*–*E*). The staining was stronger along the acinus wall (fig. 1). The probe did not stain the eggs (fig. 1*F*), in agreement with the absence of M-type mtDNA from all the egg samples tested so far (Ghiselli et al. 2011).

**Fig. 2.—:**
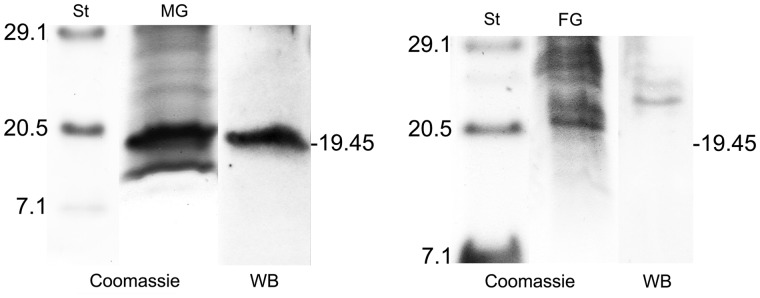
RPHM21 detection by Western blot. Left: broad-range protein standards (St), SDS-PAGE of male gonadic extract (MG) stained with Coomassie blue, and Western blot with anti-RPHM21 showing a band of about 20 kDa. On the right: broad-range protein standards (St), SDS-PAGE of female gonadic extract (FG) stained with Coomassie blue, and Western blot with anti-RPHM21 in which no band of 20 kDa is detected.

Immunoblotting of gonadic extracts was performed using an antibody (anti-RPHM21) generated against peptides synthesized from the predicted amino acid sequence in the C-terminus of the protein (two adjacent peptides of 17 and 16 aa). In male gonadic extracts, the Western blot showed a band of ∼20 kDa (fig. 2). Actually, 19.45 kDa is the molecular weight of RPHM21 estimated from the amino acid sequences. No band of the corresponding molecular weight was detected by Western blot of female gonadic extracts (fig. 2).

**Fig. 3.—:**
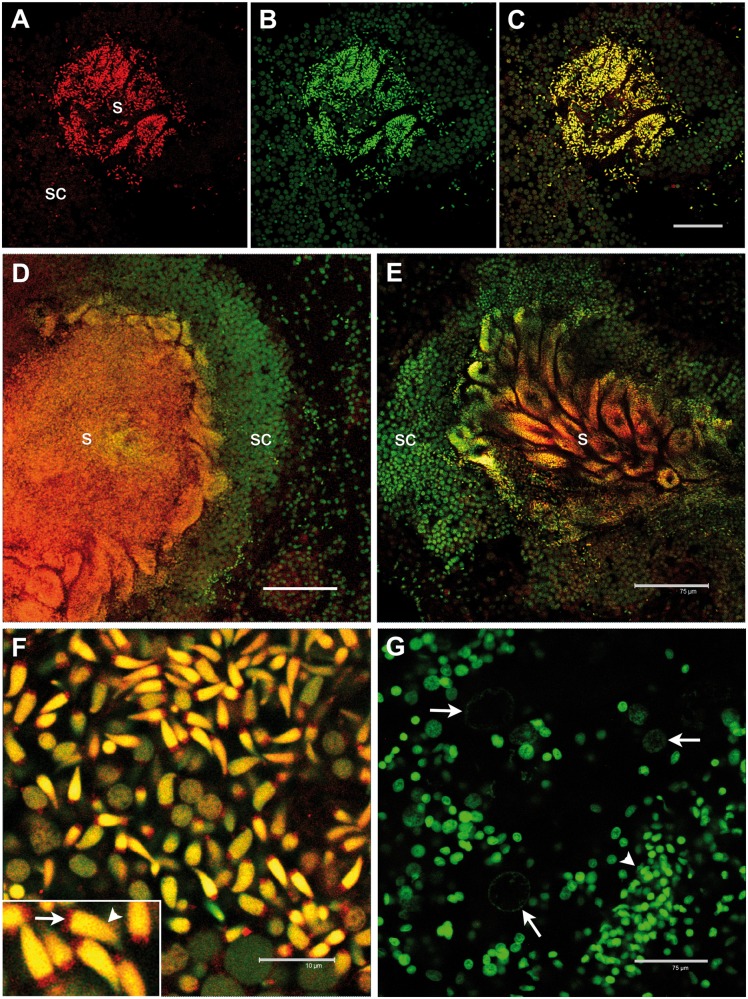
RPHM21 protein localization in *Ruditapes philippinarum* testis. (*A*) RPHM21 antibody staining (in red) on a whole male acinus section (s: mature spermatozoa; sc: spermatogenic cells); (*B*) the same section stained with the nuclear dye TO-PRO3 (in green); (*C*) merge of (*A*) and (*B*) sections showing a colocalization (in yellow) of the two labeling; (*D*, *E*) anti-RPHM21 antibody strongly labeled mature spermatozoa (s) in the acinus lumen with the staining present in both mitochondria and the nucleus, while no strong reaction is detected in spermatogenic cells (sc), whose nuclei are visible in green (TO-PRO3); (*F*) a higher magnification of mature spermatozoa clearly shows RPHM21 staining in sperm mitochondria (arrow) and the colocalization of RPHM21 with nuclear material in the sperm heads (arrowhead); (*G*) in eggs (with big and faint nuclei; arrows) no RPHM21 detectable staining is present, and only nuclear material is visible (in green, TO-PRO3). The smaller nuclei of somatic cells surrounding acini are also visible (arrowhead). All gonadic sections are visualized at confocal microscope. RPHM21 antibody staining in red; TO-PRO3 nuclear dye in green. Scale bars: (*A–E*) and (*G*) = 75 µm; (*F*) = 10 µm, inset: same scale bar as (*F*) corresponds to 4 µm.

Only male gonadic tissue showed a staining with anti-RPHM21. The staining was localized in spermatogenic cells, with a deeper labeling in mature spermatozoa, both in mitochondria (in the four mitochondria of the spermatozoon midpiece) and in the nucleus (fig. 3). No staining was visible in surrounding tissues (connective and intestine) or in eggs (fig. 3*G*).

**Fig. 4.—:**
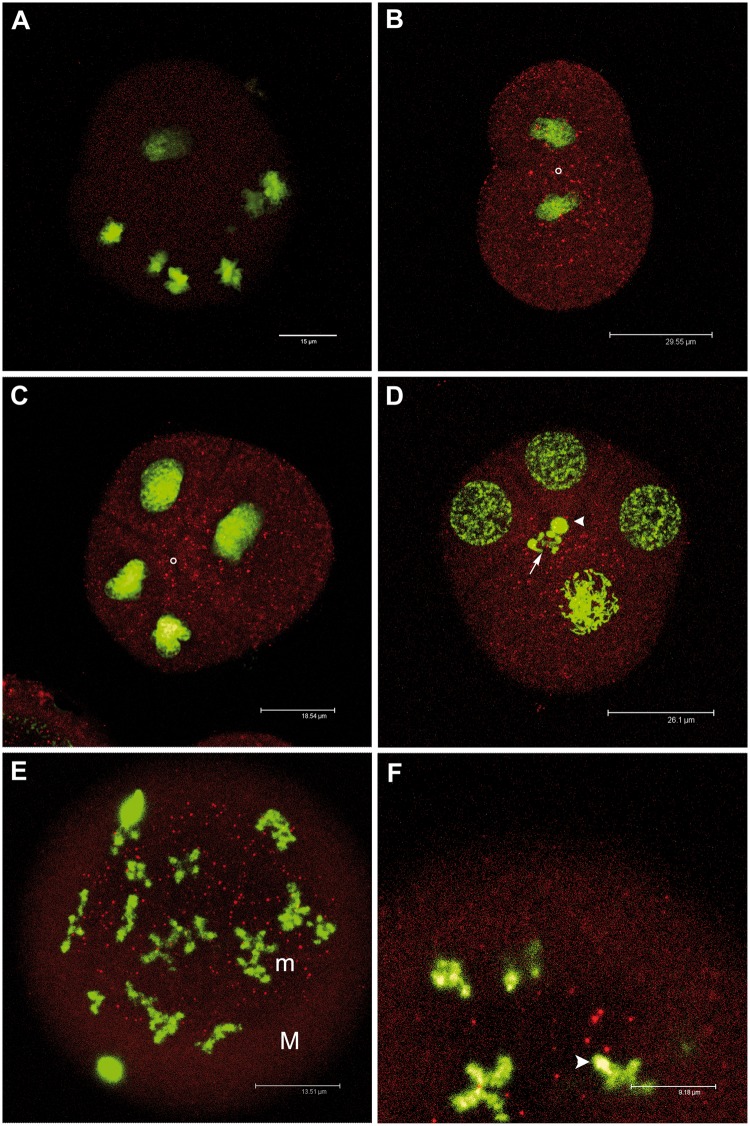
RPHM21 localization in *Ruditapes philippinarum* early embryos. (*A*) Control sample: eight-blastomere embryo in which the first antibody (anti-RPHM21) was omitted; no staining is detected. Nuclei in green (TO-PRO3); (*B*) two-blastomere embryo showing a spotted staining of RPHM21 (in red) more concentrated in the region flanking the cleavage between the two blastomere (the animal–vegetal axis is indicated by a white circle) and around nuclei; (*C*) four-blastomere embryo showing a spotted staining around the animal–vegetal axis (indicated by a white circle); (*D*) RPHM21 localization in a four-blastomere embryo in which the biggest blastomere (the D one) is shown shortly before the formation of the following segmentation furrow that will form micromere 1d and macromere 1D of the eight-blastomere stage. The future position of micromere 1d (its nucleus is indicated by an arrow) will be in proximity to the animal–vegetal axis where also the nucleus of the polar body is visible (arrowhead). It is evident that micromere 1d will take up most of the cytoplasmic region containing the labeled spots; (*E*) in 32-blastomere embryos bigger immunostained spots are localized only in micromeres (M = macromeres, m = micromeres); (*F*) a detail of the 32-blastomere embryo showing some RPHM21 spots surrounding a micromere nucleus (arrowhead). RPHM21 antibody staining in red; TO-PRO3 nuclear dye in green. All the embryos are visualized (at confocal microscope) along *Z* axis, that coincides to the animal–vegetal axis direction.

Some of the embryos of the two matings showed a spotted staining, while others did not show any staining. The spot number and dimension appeared to increase from the two-blastomere stage to the 32-cell embryo. Four- and eight-cell embryos had a deep staining localized around the animal–vegetal axis (fig. 4). In 32-blastomere embryos, the staining was localized in micromeres. No staining was present in control embryos (fig. 4*A*).

### Function and Structure of RPHM21

**Fig. 5.—:**
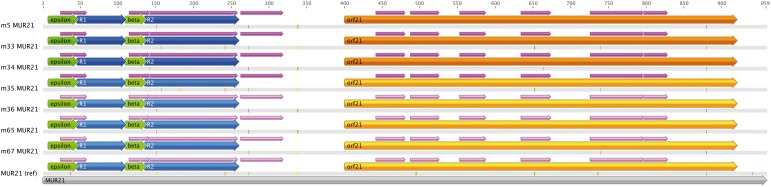
Male unassigned region 21 (MUR21) regions. Upstream, the repeat region containing both repeats (R1 and R2) and spacers (ε and β). Downstream, *orf21* (in orange). Domains similar to viral sequences (BlastN) are reported in pink.

The analysis of the whole MUR21 nucleotide sequence with BlastN revealed in *orf21* one significant similarity with Bacteriophage phi-C31 complete genome (table 1). On the whole, 44 hits of phages and viruses were detected in MUR21, mainly in *orf21* sequence and in the repeat region upstream *orf21* (table 1 and fig. 5).

**Table 1:** MUR21 Nucleotide Sequence Analysis with BlastN

Blastn Hits	Protein	Sequence ID	Identities	Gaps	Query (bp)	MUR21 (bp)
Bacteriophage phi-C31 complete genome		emb|AJ006589.3	33/37 (89%)	0/37 (0%)	636–672	*orf21*
Acanthamoeba polyphaga moumouvirus, complete genome		gb|JX962719.1	36/43 (84%)	3/43 (6%)	160–203	R2
Clostridium phage phiZP2, complete genome		gb|JQ729992.1	27/30 (90%)	0/30 (0%)	798–827	*orf21*
Haemophilus influenzae R2866, complete genome	Pantothenate kinase	gb|CP002277.1	36/44 (82%)	1/44 (2%)	160–203	R2
Lymphocystis disease virus - isolate China, complete genome		gb|AY380826.1	25/27 (93%)	0/27 (0%)	186–212	R2
Canarypox virus strain ATCC VR-111, complete genome		gb|AY318871.1	54/72 (75%)	5/72 (6%)	136–204	R2
Paramecium bursaria Chlorella virus MA-1E, partial genome		gb|JX997173.1	30/36 (83%)	0/36 (0%)	489–524	*orf21*
Paramecium bursaria Chlorella virus CvsA1, partial genome		gb|JX997165.1	30/36 (83%)	0/36 (0%)	489–524	*orf21*
Paramecium bursaria Chlorella virus CviKI, partial genome		gb|JX997162.1	30/36 (83%)	0/36 (0%)	489–524	*orf21*
Megavirus courdo7 isolate Mv13-c7, partial genome		gb|JN885992.1	30/36 (83%)	0/36 (0%)	160–195	R2
Cafeteria roenbergensis virus BV-PW1, complete genome		gb|GU244497.1	30/36 (83%)	0/36 (0%)	160–195	R2
Brachyspira hyodysenteriae WA1, complete genome	Hypothetical protein	gb|CP001357.1	76/107 (71%)	14/107 (13%)	160–257	R2
Deerpox virus W-1170-84, complete genome		gb|AY689437.1	26/28 (93%)	1/28 (3%)	176–202	R2
Bacteriophage Phi JL001, complete genome		gb|AY576273.1	23/24 (96%)	0/24 (0%)	635–658	*orf21*
Paramecium bursaria Chlorella virus NE-JV-1, partial genome		gb|JX997176.1	28/33 (85%)	0/33 (0%)	554–586	*orf21*
Megavirus lba isolate LBA111, complete genome		gb|JX885207.1	26/30 (87%)	0/30 (0%)	166–195	R2
Megavirus courdo11, complete genome	Putative ankyrin repeat protein	gb|JX975216.1	26/30 (87%)	0/30 (0%)	166–195	R2
Abalone herpesvirus Victoria/AUS/2009, complete genome		gb|JX453331.1	24/25 (96%)	1/25 (4%)	638–661	*orf21*
Human adenovirus 55 isolate CQ-2903, complete genome		gb|JX123029.1	23/25 (92%)	0/25 (0%)	116–140	β/R2
Human adenovirus 55 isolate CQ-814, complete genome		gb|JX123027.1	23/25 (92%)	0/25 (0%)	116–140	β/R2
Pseudomonas phage PA7, partial genome		gb|JX233784.1	23/25 (92%)	0/25 (0%)	284–308	Interspace*
Bacillus phage BtCS33, complete genome		gb|JN191664.1	20/20 (100%)	0/20 (0%)	298–317	Interspace*
Pseudomonas phage Lu11, complete genome		gb|JQ768459.1	28/33 (85%)	0/33 (0%)	638–670	*orf21*
Clostridium phage phi8074-B1, complete genome		gb|JQ246028.1	25/27 (93%)	1/27 (3%)	740–765	*orf21*
Elephant endotheliotropic herpesvirus 2		gb|JQ300037.1	28/33 (85%)	0/33 (0%)	25–57	ε/R1
Macaca fuscata rhadinovirus isolate 12E2, complete genome		gb|JN885137.1	35/45 (78%)	0/45 (0%)	264–308	Interspace*
Macaca fuscata rhadinovirus isolate 3A1, complete genome		gb|JN885136.1	35/45 (78%)	0/45 (0%)	264–308	Interspace*
Bacillus phage phIS3501, complete genome		gb|JQ062992.1	20/20 (100%)	0/20 (0%)	298–317	Interspace*
Elephant endotheliotropic herpesvirus 2		gb|HM568561.2	28/33 (85%)	0/33 (0%)	25–57	ε/R1
Megavirus chiliensis, complete genome	Putative ankyrin repeat protein	gb|JN258408.1	26/30 (87%)	0/30 (0%)	166–195	R2
Ariquemes virus segment M, complete sequence		gb|HM119405.1	20/20 (100%)	0/20 (0%)	745–764	*orf21*
Abalone herpesvirus Victoria/AUS/2007 p073c gene, complete cds		gb|HQ400678.1	24/25 (96%)	1/25 (4%)	638–661	*orf21*
Human adenovirus 55 strain QS-DLL, complete genome		gb|FJ643676.1	23/25 (92%)	0/25 (0%)	116–140	β/R2
Streptococcus phage P9, complete genome		gb|DQ864624.1	28/33 (85%)	0/33 (0%)	184–216	R2
Goatpox virus G20-LKV, complete genome		gb|AY077836.1	37/46 (80%)	3/46 (6%)	727–769	*orf21*
Goatpox virus Pellor, complete genome		gb|AY077835.1	37/46 (80%)	3/46 (6%)	727–769	*orf21*
Goatpox virus strain GT4-STV42-72, complete cds	Thymidine kinase gene	gb|AY773087.1	37/46 (80%)	3/46 (6%)	727–769	*orf21*
Camelpox virus M-96 from Kazakhstan, complete genome		gb|AF438165.1	26/30 (87%)	0/30 (0%)	766–795	*orf21*
Mirabilis mosaic virus, complete genome		gb|AF454635.1	31/37 (84%)	2/37 (5%)	443–479	*orf21*
Camelpox virus CMS, complete genome		gb|AY009089.1	26/30 (87%)	0/30 (0%)	766–795	*orf21*
Pseudomonas phage phiKZ, complete genome		gb|AF399011.1	23/25 (92%)	0/25 (0%)	284–308	Interspace*
Macaca fuscata rhadinovirus, complete genome		gb|AY528864.1	35/45 (78%)	0/45 (0%)	264–308	Interspace*
Macaca mulatta rhadinovirus 17577, complete genome		gb|AF083501.3	35/45 (78%)	0/45 (0%)	264–308	Interspace*
Macaca mulatta rhadinovirus 26-95 L-DNA, complete sequence		gb|AF210726.1	35/45 (78%)	0/45 (0%)	264–308	Interspace*

Note.—On the whole, 44 hits of phages and viruses were detected in MUR21, mainly in *orf21* sequence and in the repeat region upstream *orf21.*

RPHM21 analysis with HMMER showed one significant hit (E-value = 0.05), that is *Cydia pomonella* granulosis virus (Baculoviridae, dsDNA virus) ORF140 similar to XcGV ORF178 (containing a domain similar to Fibroblast growth factor). The other three hits found were as follows: a Zinc-binding protein (*Drosophila melanogaster*) (E-value = 0.33); Autophagy protein Apg17 (*Dictyostelium discoideum*) (E-value = 0.59); ABC-2 family transporter protein (*Thermofilum pendens* Hrk 5) (E-value = 0.9). Despite the nonsignificant E-values (HMMER E-values of 0.1 or less are significant; see HMMER User Guide Version 2.2, page 9), all these three hits are supported by other in silico analyses (Milani, Ghiselli, Guerra, et al. 2013). We also would like to point out that the E-value (which measures the number of hits that are expected to be found by chance when searching a database) depends strongly on two parameters: the database size and the sequence length. The larger the database and the shorter the query sequence, the greater the chance to find a match by chance. As we are dealing with short sequences searched in quite large databases, it is possible that nonsignificant but borderline results as those reported are actually reliable.

**Fig. 6.—:**
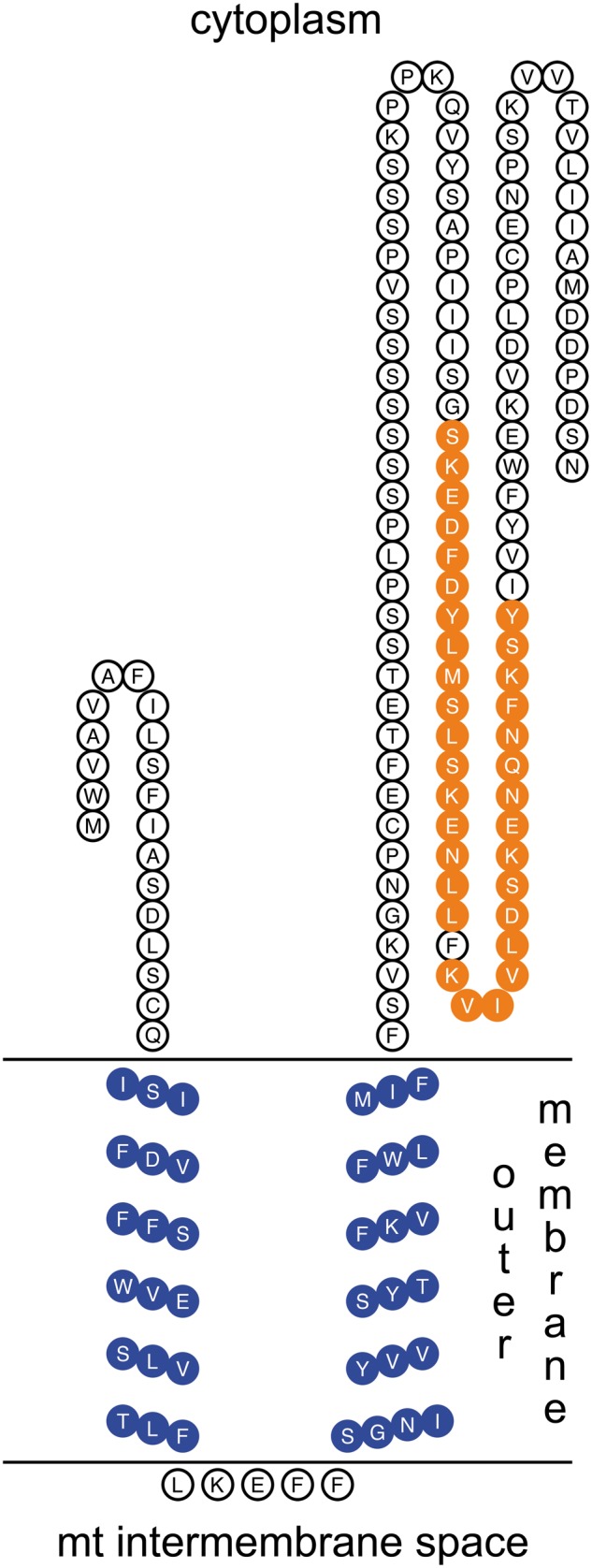
A proposed localization for RPHM21. The N-terminus is localized in the cytoplasm, followed by the two TMHs (in blue) inside the mitochondrial outer membrane, and by the long C-terminus that is also localized in the cytoplasmic side of the membrane. The synthesized antigenic peptides used to generate antibodies are in orange.

FUGUE found two hits with high confidence: the first is ankyrin repeat domain of trpv1 (transient receptor potential cation channel) (*Z* score ≥ 4.0, 95% confidence), and the other hit is sprpn10 vwa domain (26s proteasome regulatory subunit rpn10) (*Z* score ≥ 3.5, 90% confidence), a ubiquitin interacting motif. ProTeus found 1 group with signature FIL: electron transport GO biological process, supporting some kind of membrane association. PredictProtein predicted the presence of two TMHs (20–37 and 43–61 residues). Mitochondria were indicated as possible subcellular localization (LOCtree implemented in PredictProtein: reliability index = 6 over 9; values ≥4 denote good predictions), and also the nucleus, even if with a low prediction score (LOCtree2 prediction: score = 17 over 100). The proposed RPHM21 position/orientation is reported in figure 6. According to the protein secondary structures predicted with Quick2D, both RPHM21 and the ubiquitin ligase MK3 (protein resulted similar to RPHM21) of Murid herpesvirus 4 showed two TMHs and a cytoplasmic C-terminus (supplementary figs. S1 and S2, Supplementary Material online).

## Discussion

### Localization of *orf21* Transcript and Its Protein RPHM21

The Manila clam *R. philippinarum* is strictly gonochoric and its gonad forms every year at the beginning of the spawning season, after which it is degraded (Devauchelle 1990). The gonad includes strongly branching acini made up of germinative epithelium supported by connective tissue (fig. 1*F*). Gonia localize at the periphery of the acinus, while mature gametes are free in the acinus lumen (Devauchelle 1990). ISH performed with *orf21* antisense riboprobe showed a specific transcription in the acinus and its lumen, indicating a positive reaction with both spermatogenic cells and mature spermatozoa (fig. 1): the staining was present in spermatogenic cells when the acinus lumen was empty, while it was restricted to spermatozoa when they filled the lumen. *orf21* showed an high staining also along the acinus wall, the acinus border formed of undifferentiated germinal cells (fig. 1*A* and *B*). The observation that the transcript was present in the acinus wall points to an early transcription, since the first stage of sexual cell differentiation. When spermatogenesis arrests, *orf21* transcription in immature germinal cells stops, but *orf21* transcripts are still visible in the acinus lumen, meaning that they are stored in mature sperm. As expected, female tissues were not stained, being *orf21* specific of the M-mtDNA, which is commonly absent from them (Ghiselli et al. 2011).

The existence of an *orf21* product (RPHM21 protein) in male gonads was verified by the presence in the Western blot of a band of about 20 kDa (fig. 2), consistent with the estimated molecular weight of RPHM21 (19.45 kDa). RPHM21 was expressed in male germ cells, while it was absent in female germ cells, since no RPHM21 band was detected. Moreover, RPHM21 protein immunolocalization showed that male germ cells were specifically stained with anti-RPHM21 (fig. 3), on the contrary, no staining was detected in eggs (fig. 3*G*). The staining was localized in spermatogenetic cells, with a deeper labeling in mature spermatozoa, both in mitochondria and nucleus (fig. 3*F*). Therefore, *orf21* is highly transcribed since the first stage of male sexual cell differentiation (acinus wall cells, i.e., spermatogonia) (fig. 1), but only in the following stages (moving toward the acinus lumen) the transcripts are translated producing RPHM21 (fig. 3). In mature sperm, both transcript and the protein were detectable (figs. 1 and 3).

This is the first time a lineage-specific mitochondrial protein is immunodetected in DUI early embryos. Overall, the spot number and dimension in the stained embryos increased from the two-blastomere stage to the 32-cell embryo (fig. 4). It has to be considered that RPHM21 visualized in developing embryos can be the protein amount already present in the spermatozoon (fig. 3) and imported with fertilization, plus the product of translation of *orf21* transcript stored in spermatozoa (fig. 1). Moreover, in embryos, newly transcribed *orf21* and its product may contribute to the observed staining, thus explaining the increasing of spot number and dimension from the first embryonic stages to the 32-cell embryo.

Four- and eight-cell embryos showed a deep staining around the animal–vegetal axis (fig. 4). We already showed that germ line determinants are localized in the same embryonic area (Milani et al. 2011), so the positioning of RPHM21 in the same region and its increasing amount during embryo growth supports a role in development. In 32-blastomere embryos, the staining was localized in micromeres (fig. 4). The presence of RPHM21 in all analyzed embryos may appear to be in contrast with its male-specific activity, as supported by our observations in adult tissues. Nevertheless, two considerations have to be done. First, different sex-specific post translational regulation of RPHM21 might be involved in early developmental stages: as discussed in Milani, Ghiselli, Nuzhdin, et al. (2013) main differences between males and females are likely to be quantitative rather than qualitative, since generally there are thresholds to be exceeded to have effects or changes. Second, the presence of RPHM21 in male and female embryos could also have different outcomes because of different epistatic interactions with sex-specific products. In the present work, we had no chance to know the sex of analyzed embryos, because genetic markers of sex are not available. Nonetheless, a sex ratio ranging from 100% females to 90% males was observed in DUI progenies (see Zouros 2013 for a review). The production of sex-biased families for *R. philippinarum* will allow us to predict the sex of the offspring and check for differential RPHM21 transcription and expression during embryo development and eventually find at what age the differentiation takes place.

### Viral Origin of *orf21*

Recently, in silico analyses of mitochondrial ORFans suggested their possible origin through viral sequence endogenization (Milani, Ghiselli, Guerra, et al. 2013). One clue pointing in that direction was the identification of CRISPR-associated sequences (Cas) in two of them: *R. philippinarum* MORF and *Paphia euglipta* ORF (Milani, Ghiselli, Guerra, et al. 2013). Clustered regularly interspaced short palindromic repeats (CRISPR) are a family of DNA direct repeats separated by nonrepetitive spacers of regular size. CRISPR are found in most bacterial and archaeal genomes and seem to provide resistance against mobile genetic elements (Jansen et al. 2002; Bolotin et al. 2004, 2005; Mojica et al. 2005; Makarova et al. 2006; Barrangou et al. 2007): after viral infection, hosts integrate CRISPR in their genome, enhancing the resistance to phage infection probably via RNA interference (Bolotin et al. 2005). Cas proteins show similarity to helicases and repair proteins (Pourcel et al. 2005) and are thought to be involved in the propagation and functioning of CRISPR (Bolotin et al. 2005). Interestingly, in *R. philippinarum,* the region upstream *orf21* is characterized by repeats that, as CRISPRs, are separated by spacers (motifs ε and β, of 28 and 26 bp, respectively; see fig. 5 and Ghiselli et al. 2013). Although other motifs found in *R. philippinarum* mtDNA intergenic regions showed homology with motifs related to mtDNA replication and transcription, all the performed analyses failed to identify any similarity of ε and β with known motifs (Ghiselli et al. 2013).

BlastN analysis showed many hits from phages and viruses in MUR21, mainly in the *orf21* and in the repeat region (table 1 and fig. 5). Another clue in favor of the hypothesis of a viral origin of RPHM21 comes from the analysis performed with HMMER that retrieved a significant hit with a mitogen (fibroblast growth factor, FGF; i.e., a factor that triggers mitosis) of a baculovirus. FGFs are fundamental for the processes of proliferation and differentiation in a wide variety of cells and tissues, and are involved in embryonic development, cell growth and differentiation, and morphogenesis. Overall, the finding of multiple virus-related hits and the presence of some features that could be ascribable to a CRISPR/Cas system further support a viral origin of *orf21*.

### Putative Function of RPHM21

Given the absence of related proteins in databases, the function of RPHM21 has to be inferred from the presence of structural motifs showing similarities with known domains. Considering previous data (Milani, Ghiselli, Guerra, et al. 2013) and the data presented here, we can summarize RPHM21 main putative features: 1) the presence of two TMHs; 2) a possible viral origin; 3) the presence of domains involved in immune system and cell cycle regulation; 4) a binding site for ubiquitin, and 5) the presence of domains involved in cytoskeleton interactions. Given these premises, we found a striking similarity between RPHM21 and MK3, a modulator of immune recognition (MIR), which is a viral protein involved in the immune recognition pathway (Coscoy and Ganem 2003). Many viruses have evolved strategies for evading T-cell-mediated host immunity by preventing the display of surface molecules that are recognized by the immune system (Coscoy and Ganem 2003). Many viral regulators cause the ubiquitination of these surface molecules, controlling the trafficking and/or the direct degradation of their targets. MIRs and homolog proteins are involved in such mechanisms functioning as ubiquitin ligases. The proteins of the ubiquitination machinery comprise several ubiquitin-like proteins and hundreds of ubiquitin-conjugating enzymes. Ubiquitin is mainly known for its role as a tag that induces protein degradation either by the proteasome or through targeting to lysosomes, but it is well established that ubiquitination is also a component of most cellular signaling pathways for the control of cell adhesion, polarity and directional migration (Schaefer et al. 2012). Most of the in silico predicted functions of RPHM21 such as reorganization of cytoskeleton, cell migration, cell cycle control, chromatin remodeling, and transcriptional control are shared with MK3 (Ronkina et al. 2008; Milani, Ghiselli, Guerra, et al. 2013). Moreover, the two proteins show structure similarities (fig. 6; supplementary figs. S1 and S2, Supplementary Material online): as predicted for RPHM21, MIRs present two putative transmembrane domains and N- and C-terminus both arranged on the cytosolic side of the membrane they are associated with (Sanchez et al. 2002; Coscoy and Ganem 2003). Nonetheless, the fact that RPHM21 is localized in both mitochondria and nucleus of spermatozoa agrees with the wide distribution that characterizes many of the ubiquitination proteins cited, as well as MIRs (Kurz et al. 2002; Ronkina et al. 2007, 2008).

All that considered, RPHM21 is similar to ubiquitination-related proteins that participate in cytoskeletal organization, transcription, cell fate determination, differentiation, proliferation and migration, and to viral proteins involved in the immune recognition pathway acting as ubiquitin ligases.

### Role of RPHM21 in Mitochondrial Inheritance and Sex Determination

As shown in previous studies (Cao et al. 2004; Obata and Komaru 2005; Cogswell et al. 2006; Milani et al. 2011, 2012), during the development of DUI male embryos, spermatozoon mitochondria show a peculiar distribution, the aggregated pattern, which is absent in female embryos and in species lacking DUI. This pattern is most likely the result of specific interactions between sperm mitochondria and cytoskeleton by which they are actively transferred to the PGCs, where they become dominant during male germ line formation. It was proposed that the midbody derived from the central spindle of the first embryonic division is involved in positioning the aggregate of spermatozoon mitochondria in the middle of the first cleavage furrow together with germ line determinants (Milani et al. 2011). Given RPHM21 predicted functions and its localization in sperm mitochondria and around the animal–vegetal axis of embryos (figs. 3 and 4), and given the capability of viral proteins to interfere with mitochondrial positioning altering cytoskeleton conformation (Doorbar et al. 1991; Galluzzi et al. 2008; Ohta and Nishiyama 2011), we suggest an involvement of this novel mitochondrial protein in the interactions with specific motor proteins that would carry sperm mitochondria to the central spindle, where the cleavage furrow of the two-cell stage forms. We propose that RPHM21 prevents the recognition of male mitochondria by the degradation machinery, allowing their survival in the zygote. The process might involve a mechanism similar to that of MIRs, including the detach of specific tags associated with spermatozoon mitochondria, that normally lead to their degradation by the ubiquitination machinery. Moreover, RPHM21 cytoskeleton-binding domains support a role in the distribution pattern of spermatozoon mitochondria observed in early DUI embryos. The outcome of the interactions between RPHM21 and the cytoskeleton would depend on the abundance, in the zygote, of nuclear-encoded factors which would determine the fate of sperm mitochondria (Milani, Ghiselli, Nuzhdin, et al. 2013).

The tight association between sex and type of mitochondrial inheritance in DUI organisms raised the question about the causality of this relationship: Are M-type mitochondria responsible for inducing spermatogenesis (therefore inducing maleness)? There is no clear-cut evidence that can answer this question, actually different studies reached opposite conclusions. On the one hand, the presence of DUI (and specifically the presence of a functional genome-specific mitochondrial ORFan) appears to be strictly related to gonochorism in freshwater mussels (Bivalvia Unionidae). In fact, Breton et al. (2011a) found DUI in all the analyzed gonochoric species, while it was missing in all the hermaphrodites, in which the ORFs showed heterogeneous macromutations. On the contrary, Kenchington et al. (2009) proposed that M-type mtDNA inheritance and maleness are not causally linked in marine mussels (Bivalvia Mytilidae). The matter still remains unsolved, but if we want to confirm or rule out the possibility that M-type mitochondria have a role in gonad differentiation, we need to keep investigating the functional and structural differences between M and F-type mtDNAs, among which their specific ORFans. Several DUI models based on breeding experiments in *Mytilus* (Diz et al. 2013; Zouros 2013) proposed that DUI phenotypes in the progeny (i.e., sex, distribution patterns of sperm mitochondria and presence/absence of M-type mtDNA) are controlled by the maternal genotype. A reanalysis of sex-determination models in *Mytilus* (Yusa et al. 2013) confirmed the maternal effects hypothesis, but also predicted the existence of secondary factors inherited from both parents: such minor sex-determining factors could be nuclear or mitochondrial. Accordingly, transcriptomic differences found in gonads of *R. philippinarum* males belonging to families with opposite sex-bias lead to the hypothesis that also some factors carried by the spermatozoon could be involved in the determination of DUI phenotypes (Ghiselli et al. 2012). Some of these factors could be nuclear-encoded (Milani, Ghiselli, Nuzhdin, et al. 2013), but the best candidate for a possible mitochondrial factor is RPHM21. Supposing that it was a masculinizing factor, sperm from different males might carry different amount of transcript and/or protein, determining the different quantity of protein in the embryo thus shifting its development toward maleness.

### Viral Protein Co-Option

The inferred functional features of RPHM21 and its increasing amount in *R. philippinarum* early embryos (fig. 4) are consistent with a possible role for this protein during embryo development. Indeed, RPHM21 showed similarities with proteins involved in transcription activation and chromatin modification related to cell cycle, cytoskeleton and centrosome organization, spindle orientation, cell proliferation, migration and differentiation, pointing also to a role in intercellular signaling during development (Milani, Ghiselli, Guerra, et al. 2013). Also, a viral origin of this paternally transmitted mitochondrial gene could support the hypothesis of a developmental role: some integrated viral elements are involved in the early development of their host, as an endogenous retrovirus recently reported to be the earliest expressed gene detected following fertilization in mouse (Kigami et al. 2003; Kageyama et al. 2004). Another example of retroviral gene co-opted by the host with a role in early development is syncytin, a retroviral envelope proteins present in mammals (Mi et al. 2000). This viral gene is indispensable for maternal–fetal exchange, tissue remodeling during placental development and protecting the developing fetus from the maternal immune response (Cross et al. 1996; Lala and Hamilton 1996; Munn et al. 1998). Other retroviral-related genes are expressed in germ cells: some proteins of viral origin expressed predominantly in the testis, and localized in discrete dots in the nucleoplasm, seem to have a role during sperm differentiation (Inoue et al. 2000), and an infection by a vertically transmissible virus is the cause of masculinization (conversion of secondary sexual characters) of female isopods (Oniscoidea, Crustacea) (Juchault et al. 1991). Several hits regarding M-type mtDNA-specific ORFs point to a role in spermatogenesis and reproduction (Milani, Ghiselli, Guerra, et al. 2013): these hits might indicate a co-option of the novel proteins in sex-specific functions. In this concern, it was proposed that marine viruses manipulate life cycle and reproduction of their metazoan hosts, as, for example, in the sea slug *Elysia chlorotica* (Gastropoda, Plakobranchidae), in which endogenous and vertically transmitted retroviruses were suggested to be partly responsible for the slug reproductive cycle (Pierce et al. 1999).

## Conclusions

It is quite clear that in DUI organisms sperm mitochondria evolved the ability to escape the degradation mechanism of the zygote and invade the germ line. Although such ability is well documented, whether M-type mitochondria play a role in gonad development and/or spermatogenesis is still unknown. The mitochondrial ORFans could be responsible for or participate in the DUI mechanism and a viral origin could well explain the acquired capability of M-type mitochondria to avoid the degradation, enter the germ line and proliferate, that is what viruses do best: to elude host immune system and proliferate. However, this does not explain the association with male sex. A mitochondrion that acquires the ability to escape degradation would be strongly advantaged and would be preferentially transmitted to the progeny, through both males and females, thus quickly increasing its genotype frequency in the population and eventually reaching fixation. In this case, we would not observe DUI. One possible explanation is that M-type mtDNA germ line invasion can succeed only in males, maybe because of epistatic interactions between the factors causing DUI and maleness factors (e.g., genes with male-biased expression). Another explanation is that the endogenization of the viral protein conferred the mitochondrion also a sex-distortion capability. In this case, a nuclear restorer gene would have evolved to avoid a shift of the sex-ratio toward males (see Passamonti and Ghiselli 2009 for a detailed discussion).

The most studied DUI taxa (Mytilidae, Unionidae, and Veneridae) are evolutionary distant, so it is also plausible that DUI mechanism of action and its interactions (if any) with sex-determining genes are different (especially if DUI evolved multiple times; Milani, Ghiselli, Guerra, et al. 2013). Most likely, several maternal and nuclear factors contribute to sex determination forming a complex network, and DUI factors could intervene at different levels of the sex determination cascade, maybe leading to different effects in different taxa.

## Supplementary Material

Supplementary figures S1 and S2 are available at *Genome Biology and Evolution* online (http://www.gbe.oxfordjournals.org/).

Supplementary Data
