# Supplementary material for: Paternally Transmitted Mitochondria Express a New Gene of Potential Viral Origin
Source: Genome Biol Evol. 2014 Feb 5;6(2):391–405. doi: 10.1093/gbe/evu021 (PMC3942028; doi:10.1093/gbe/evu021)
Supplement: Supplementary Data [file supp_6_2_391__index.html]

Paternally transmitted mitochondria express a new gene of potential viral origin — Paternally Transmitted Mitochondria Express a New Gene of Potential Viral Origin — Supplementary Data 

# Paternally Transmitted Mitochondria Express a New Gene of Potential Viral Origin

## Supplementary Data

files

**Files in this Data Supplement:**

- Supplementary Data - pdf file
- Supplementary Data - pdf file
