# Supplementary material for: Paternally Transmitted Mitochondria Express a New Gene of Potential Viral Origin
Source: Genome Biol Evol. 2014 Feb 5;6(2):391–405. doi: 10.1093/gbe/evu021 (PMC3942028; doi:10.1093/gbe/evu021)

Supplemental Figure 1. Protein secondary structure prediction of RPHM21 and the ubiquitin ligase MK3. Both proteins showed two transmembrane regions. N-terminus and C-terminus “outside”. (Quick2D; http://toolkit.tuebingen.mpg.de/quick2_d).
RPHM21 (from the M-type complete mitochondrial genome, GenBank: AB065374). MK3 of Murid herpesvirus 4 (NCBI Reference Sequence: NP_044852.1).

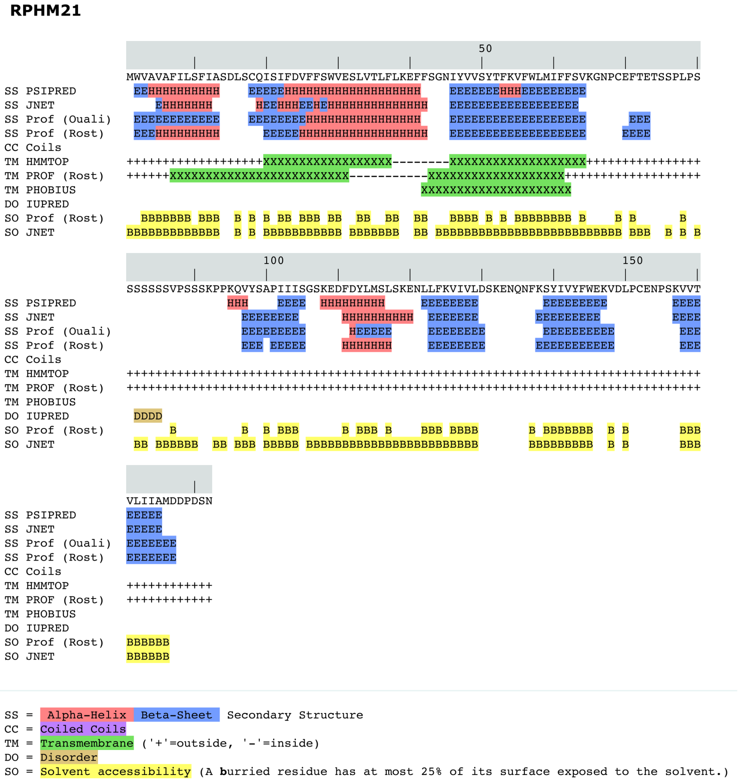

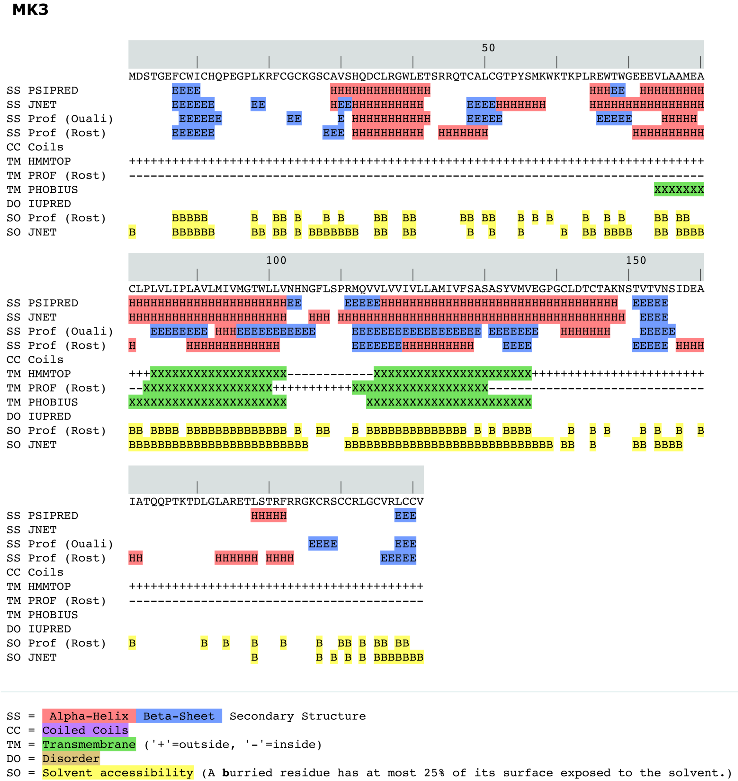

Supplement: Supplementary Data [file supp_evu021_Supp-fig1.pdf]
