# Supplementary material for: Paternally Transmitted Mitochondria Express a New Gene of Potential Viral Origin
Source: Genome Biol Evol. 2014 Feb 5;6(2):391–405. doi: 10.1093/gbe/evu021 (PMC3942028; doi:10.1093/gbe/evu021)
Supplement: Supplementary Data [file supp_evu021_Supp-fig2.pdf]

Supplemental Figure 2. Alignment of RPHM21 and the ubiquitin ligase MK3.
Both show two transmembrane regions and N-terminus and C-terminus “outside”
(http://tcoffee.crg.cat/apps/tcoffee/do:tmcoffee; Chang et al. 2012).
RPHM21 (from the M-type complete mitochondrial genome, GenBank: AB065374).
MK3 of Murid herpesvirus 4 (NCBI Reference Sequence: NP_044852.1).

12/03/13 15.46

```
T-COFFEE, Version_9.03.r1318 (2012-07-12 19:05:45 - Revision 1318 - Build 366)
Cedric Notredame

 IN  HEL  OUT

RPHM21      1 MWVAVAF----------ILSFIASDLSCQISIFDVFFSWVESLVTL----------------FL--------  38
MK3         1 MDSTGEFCWICHQPEGPLKRFCGCKGSCAVSHQDCLRGWLETSRRQTCALCGTPYSMKWKTKPLREWTWGEE  72
            1 *  :  *          :  * ... ** :*  * : .*:*:                     *          72


RPHM21     39 --------------------------------KEFFSGNIYVVSYTFKVFWLMIFFSV-------KGNPCEF  71
MK3        73 EVLAAMEACLPLVLIPLAVLMIVMGTWLLVNHNGFLSPRMQVVL-VVIVLLAMIVFSASASYVMVEGPGCLD 143
           73                                 : *:* .: **  .. *:  **.**.       :*  *   144


RPHM21     72 TETSSPLPSSSSSSSVPSSSKPPKQVYSAPIIISGSKEDFDYLMSLSKENLLFKVIVLDSKENQNFKSYIVY 143
MK3       144 TCTAKNSTVTVNSI------------DEAIATQQPT----KTDLGLARETLSTRFR--------RGKCRSCC 191
          145 * *:.  . : .*              .*    . :    .  :.*::*.*  :.         . *.     216


RPHM21    144 FWEKVDLPCENPSKVVTVLIIAMDDPDSN 172
MK3       192 RLGCVR-------------------LCCV 201
          217     *                      .  245
```
